# Ventricular Migration of Vitreous Silicone Oil Masquerading as a Ruptured Colloid Cyst

**DOI:** 10.7759/cureus.58043

**Published:** 2024-04-11

**Authors:** Nicholas S Hernandez, Anthony Nguyen, Awais Z Vance

**Affiliations:** 1 Neurosurgery, Baylor College of Medicine, Temple, USA; 2 Neurosurgery, Baylor Scott & White Medical Center-Temple, Temple, USA

**Keywords:** retinal detachment, neuroradiology, neurosurgery, intraventricular hemorrhage, silicone oils

## Abstract

The ventricular migration of vitreous silicone oil (SiO) is rare and can easily be mistaken for intraventricular hemorrhage or a ruptured colloid cyst. We report here the case of an adult male who was misdiagnosed with a ruptured colloid cyst and was subsequently found to have ventricular migration of vitreous SiO. A 57-year-old male presented unresponsive following a respiratory arrest and underwent a head computed tomography (CT) scan that demonstrated multiple ovoid hyperdensities in the ventricular system, which was concerning for a ruptured colloid cyst. He was transferred to our institution for neurosurgical evaluation.

Magnetic resonance imaging (MRI) was performed and demonstrated widespread abnormal diffusion restriction throughout the cortex and basal ganglia, consistent with anoxic brain injury secondary to hypoxic respiratory arrest. The MRI also demonstrated an abnormal signal in areas corresponding with the previously identified intraventricular lesions, which did not layer posteriorly. Given that the MRI sequence signals of the lesions in the ventricular system matched perfectly with the signals of the somewhat deflated SiO within the globe, these multiple ovoid lesions on imaging were most consistent with the migration of SiO from the vitreous body of the right globe into the ventricular system.

This case demonstrates a diagnostic error that can occur in emergent settings because of the broad differential diagnosis for cerebral ventricular hyperdensities. A ruptured colloid cyst was considered the reason for transfer, with the anticipation of neurosurgical intervention, but further imaging demonstrated that this was an incidental finding in this patient who presented in extremis. Awareness of this rare clinical condition can prevent overutilization of resources and unnecessary interventions.

## Introduction

Silicone oil (SiO) is widely used for the treatment of complex retinal detachments with reported complications including cataract formation, oil emulsification with secondary glaucoma, and subretinal migration [1]. Migration from the vitreous compartment into the optic nerve was first reported in 1983 [2]. SiO migration into the cerebral ventricles is a very rare phenomenon and was first reported in 1999 [3]. The literature most often describes this radiographic finding as incidental, and many patients are asymptomatic [4]. Symptoms are rare but include chronic headaches due to elevated intracranial pressure [5,6]. Due to the incidental nature of the findings, the time interval between SiO injection and intraventricular SiO migration is unclear, with time intervals ranging from 6.5 months to as long as 25 years [4]. On computed tomography (CT), the SiO appears as a mass-like focus of hyperdensity in the cerebral ventricles, while on magnetic resonance imaging (MRI), the focus is hyperintense to cerebrospinal fluid on pre-contrast T1-weighted images and hypointense on T2-weighted images [7]. Management of symptomatic SiO migration includes conservative medical management for pain relief and, if necessary, shunting to relieve elevated intracranial pressure [5,6].

One differential diagnosis for intraventricular SiO is a ruptured colloid cyst, which accounts for <2% of all primary brain lesions. It is typically an incidental diagnosis but can cause hydrocephalus, even presenting as sudden death [8-10]. As such, prompt, accurate diagnosis is of utmost importance [11]. 

We report here a case of a 57-year-old male patient who presented unresponsive to the hospital following a respiratory arrest. He was diagnosed at an outside facility with a presumed ruptured colloid cyst and was transferred to our neurosurgical intensive care unit (NSICU) for treatment. Subsequent imaging performed at our institution demonstrated that his intraventricular lesions were consistent with vitreous SiO migration into the cerebral ventricles.

## Case presentation

**Figure 1:**
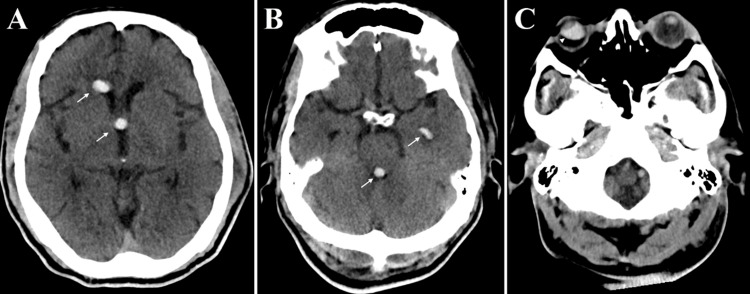
Axial slices of noncontrast head computed tomography demonstrate hyperdense ovoid lesions (white arrows) in the right frontal horn and the left temporal horn of the lateral ventricles, third ventricle, and fourth ventricle (A, B) without evidence of hydrocephalus. Imaging through the orbits shows postoperative changes of a right vitrectomy (white arrowhead, C)

A 57-year-old male patient with a history of diabetes mellitus type 2 with retinopathy, chronic congestive heart failure, and stage 5 chronic kidney disease presented to an outside facility after being found unresponsive from acute respiratory failure. He was last known well two days prior. He was found with secretions and vomitus in his nose and mouth. Chest compressions were performed for several minutes prior to the arrival of first responders. He was intubated in the field and brought to the emergency department. CT head demonstrated hyperdensities in the ventricular system which was initially diagnosed as a ruptured colloid cyst. Given these findings, he was transferred to our hospital for a higher level of care. The patient presented to our institution in an unresponsive state with metabolic acidosis. Lab work was notable for a pH of 7.1 and elevated beta-hydroxybutyrate level. CT chest demonstrated findings consistent with aspiration pneumonia, and a CT head showed multiple ovoid hyperdensities in the anterior horn of the right lateral ventricle, third ventricle, and fourth ventricle, which was interpreted as possibly reflecting a ruptured colloid cyst (Figure 1).

**Figure 2:**
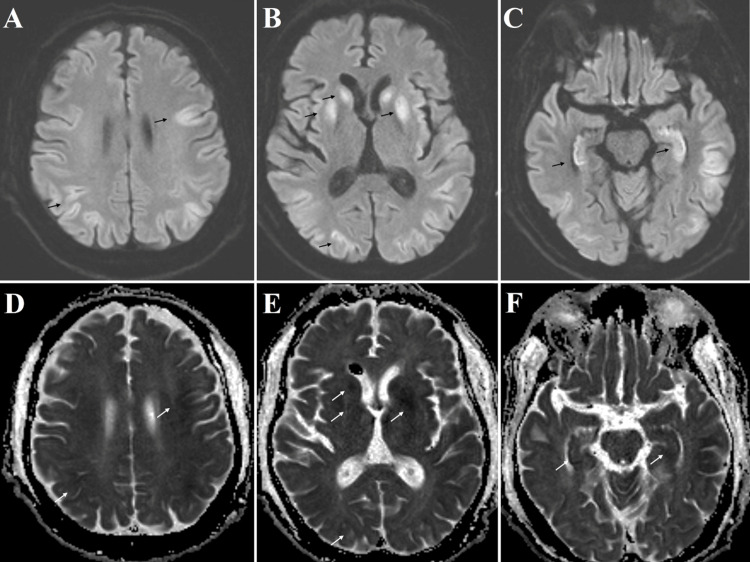
Magnetic resonance imaging with diffusion-weighted imaging (A-C) sequences and apparent diffusion coefficient maps (D-F) demonstrating numerous areas of diffusion restriction (white and black arrows) in the cortex, hippocampus, and basal ganglia consistent with anoxic brain injury

**Figure 3:**
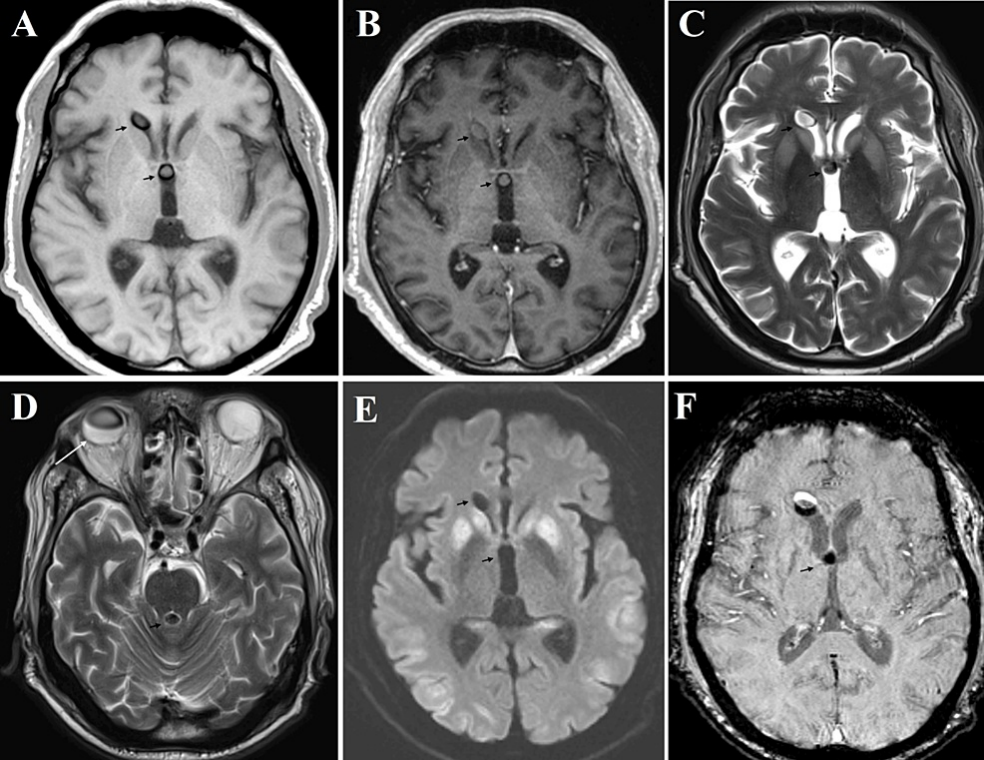
Axial slices of magnetic resonance imaging with black arrows delineating intraventricular lesions, most consistent with migrated vitreous silicone oil T1 precontrast imaging demonstrates isointense to grey matter intraventricular lesions (A) which do not contrast enhance on T1 postcontrast imaging (B). These lesions are iso- to hypointense to grey matter on T2 imaging (C, D) and do not diffusion-restrict on diffusion weight imaging (E) There is susceptibility artifact on susceptibility weighted imaging (F). Changes of a right vitrectomy are evident on T2 imaging (D)

He was transferred to the NSICU for neurosurgical evaluation and possible intervention for the presumed ruptured colloid cyst. On exam, his Glascow Coma Scale (GCS) was 7 with minimal withdrawal to pain in all extremities. He would open his eyes to pain, and his brainstem reflexes were intact. An MRI brain performed on the day of admission showed diffusely abnormal diffusion-weighted signal throughout the cortex and basal ganglia in keeping with anoxic brain injury (Figure 2). The MRI also demonstrated abnormal signal in areas corresponding with the previously identified intraventricular lesions (Figure 3). Given that the MRI sequences of the lesions in the ventricular system matched perfectly with the sequences of the somewhat deflated SiO within the globe, these multiple ovoid lesions on imaging were most consistent with migration of SiO from the vitreous body of the right globe into the ventricular system. These findings were incidental and unrelated to the patient’s respiratory arrest and subsequent anoxic brain injury.

His neurologic status did not improve. A continuous electroencephalogram (EEG) was consistent with severe anoxic brain injury. After goals-of-care discussions with the family, they elected to transition to comfort care, and the patient passed away.

## Discussion

Correct diagnosis of intraventricular migration of vitreous SiO can reduce unnecessary examinations and interventions, as seen in this case. Because of the similar appearance on CT scans, it can be misdiagnosed as other clinical entities. For example, Potts et al. and Dababneh et al. described cases of intraventricular migration of SiO that were misdiagnosed as intraventricular hemorrhage [12,13].

SiO has a high surface tension, so it will always appear as a uniform spherical high-density lesion in the ventricle on the head CT [4]. The density on CT is often greater than 85 Hounsfield units (HU) but can be as low as 50 HU. This is within the range of blood, so HU may not be the best criterion for differentiating SiO from hemorrhage. The density of SiO is lower than that of the cerebrospinal fluid, so the lesion is often detected at the top of the ventricles and can move as the patient changes position. Although not used in this case, this information can be critical as the prone examination position can be used to confirm the diagnosis of SiO migration [4,7,14,15]. Similar features have not been observed in other intraventricular lesions [2]. Although intraventricular hemorrhages often demonstrate dependency and layers within the ventricular system, its relatively higher density causes it to end up on the opposite side of where SiO would be expected to be.

As ventricular SiO migration is asymptomatic in the vast majority of cases, no treatment is required. In the uncommon scenario that treatment is required, Mazzeo et al. and Hruby et al. describe different strategies for treatment: Mazzeo et al. describing treatment consisting of administration of oral analgesia with metamizole and Hruby et al. describing treatment with ventricular peritoneal shunt to relieve increased intracranial pressure [5,6].

Important limitations to note are that this is a single reported case and that the cause for the patient's respiratory arrest was not identified. However, the most likely outcome of ventricular obstruction would have been expected to result in hydrocephalus and herniation. This would have caused possible anterior cerebral artery (ACA) distribution infarcts or irreversible tonsillar herniation. In this patient's case, diffusion restriction was primarily located in the cortices, basal ganglia, and hippocampus, suggestive of a different, unidentified cause for the patient's respiratory arrest. Thus, the ventricular migration of SiO in this case is likely an incidental finding.

## Conclusions

We demonstrate here the case of a patient who presented in extremis and was found on imaging to have intraventricular hyperdensities initially concerning a ruptured colloid cyst. However, subsequent imaging revealed that this was intraventricular migration of vitreous SiO and was an incidental finding unrelated to the patient’s presentation. The differential diagnosis of cerebral intraventricular hyperdensities is broad and includes life-threatening conditions such as ruptured colloid cysts and intraventricular hemorrhage, which can lead to acute hydrocephalus. Intraventricular migration of SiO is a rare clinical entity of which practitioners should be aware.
